# Psychological, cultural and neuroendocrine profiles of risk for preterm birth

**DOI:** 10.1186/s12884-015-0640-y

**Published:** 2015-09-03

**Authors:** R. Jeanne Ruiz, Alok Kumar Dwivedi, Indika Mallawaarachichi, Hector G. Balcazar, Raymond P. Stowe, Kimberly S. Ayers, Rita Pickler

**Affiliations:** 1 Texas A&M University Health Science Center College of Nursing, 8447 State Highway 47, Bryan, TX 77807-3260 USA; 2 Texas Tech University Health Sciences Center at El Paso Paul L. Foster School of Medicine, Department of Biomedical Sciences, Division of Biostatistics and Epidemiology, A3302 Biostatistical and Epidemiological Consulting Lab, 4801 Alberta Avenue, El Paso, TX 79905 USA; 3 The University of Texas School of Public Health, El Paso Regional Campus, 1100 North Stanton Suite 110, El Paso, TX 79902 USA; 4 Microgen Laboratories, 903 Texas Avenue, La Marque, TX 77568 USA; 5 Texas A&M University Health Science Center College of Nursing, 3950 N A.W. Grimes Blvd, Round Rock, TX 78665 USA; 6 The Ohio State University College of Nursing, Newton Hall, 1585 Neil Avenue, Columbus, OH 43210 USA

## Abstract

**Background:**

Preterm birth remains a major obstetrical problem and identification of risk factors for preterm birth continues to be a priority in providing adequate care. Therefore, the purpose of this study was to elucidate risk profiles for preterm birth using psychological, cultural and neuroendocrine measures.

**Methods:**

From a cross sectional study of 515 Mexican American pregnant women at 22–24 weeks gestation, a latent profile analysis of risk for preterm birth using structural equation modeling (SEM) was conducted. We determined accurate gestational age at delivery from the prenatal record and early ultrasounds. We also obtained demographic and prenatal data off of the chart, particularly for infections, obstetrical history, and medications. We measured depression (Beck Depression Inventory), mastery (Mastery scale), coping (The Brief Cope), and acculturation (Multidimensional Acculturation Scale) with reliable and valid instruments. We obtained maternal whole blood and separated it into plasma for radioimmunoassay of Corticotrophin Releasing Hormone (CRH). Delivery data was obtained from hospital medical records.

**Results:**

Using a latent profile analysis, three psychological risk profiles were identified. The “low risk” profile had a 7.7 % preterm birth rate. The “moderate risk” profile had a 12 % preterm birth rate. The “highest risk” profile had a 15.85 % preterm birth rate. The highest risk profile had double the percentage of total infections compared to the low risk profile. High CRH levels were present in the moderate and highest risk profiles.

**Conclusion:**

These risk profiles may provide a basis for screening for Mexican American women to predict risk of preterm birth, particularly after they are further validated in a prospective cohort study. Future research might include use of such an identified risk profile with targeted interventions tailored to the Hispanic culture.

## Background

Preterm birth (PTB) is a primary reason for neonatal morbidity and mortality, with serious health and monetary costs [1]. While PTB accounts for 75 % of perinatal deaths, many preterm infants survive but are at risk for long term impairments [2, 3]. While costs for PTB are approximately $26.2 billion yearly [4] and despite decades of research and current available prevention methods, very little is known about how to prevent PTB. Research to identify pregnancies at higher risk for PTB is vital to develop tailored, targeted interventions to prevent PTB occurrence; a recent Cochrane review recommended evaluation of a risk screening tool to predict PTB [5].

Minority populations bear a disproportionate burden of PTB [6], especially Hispanics, the largest and fastest growing ethnic group in the U.S. [7]. In the U.S. in 2012, Hispanics had a preterm rate of 11.5 % as compared to 16.5 % for Blacks and 10.3 % for whites. One in four preterm babies were Hispanic. The percentage of Hispanic women of childbearing age is estimated to increase 92 % by 2050 [8]. Given both the rate of PTB in Hispanics in the U.S. (11.38 %) [6, 7, 9] and the rate of low birth weight of 7 % in Hispanics nationally with the projected rate of growth, it is vital to establish factors predicting risk for PTB to improve health and lower costs.

Studies have identified three psychological factors—depression, coping, and mastery—that are individually associated with PTB [10, 11]. Much empirical evidence exists supporting the relationship between depression and PTB; however, the causes of the relationship are not clear [11–14]. The onset and duration of depression during pregnancy have been shown to have an impact on newborn physiology [15]. Worsening of maternal depression escalates the risk of PTB [11, 16]. Avoidance coping has been associated with depression among pregnant women [17]. Patterns of coping during pregnancy vary by population, degree of medical risk, age, education, income, race, and marital status of the mother [18] and also by the stage of pregnancy [19]. Both active and disengaged coping have been measured in pregnant women [20]. Active coping involves planning, use of emotional support, positive reframing, humor, acceptance and religion. Disengaged coping involves denial and avoidance. Previous work has focused on avoidance coping—denial and/or behavioral disengagement/mental disengagement from the perceived source of distress [17]. An association between avoidance coping and poor psychological well-being has been found [21, 22]. Healthy primigravidae women with lower income, less education, and were single had more avoidance coping [18]. A non-pharmacological approach to mediate the effects of depression, anxiety, and stress during pregnancy may be to improve active coping.

Mastery, a concept related to coping, is the belief that a person has control over their own behavior, can affect their own environment, and bring about results that they desire. Mastery may be considered a psychological resource, an aspect of resilience [23]. Among pregnant women, low mastery is associated with depressive symptoms and an increased risk of PTB and low birth weight (LBW) [10, 24]. Higher mastery is associated with lower perceived stress and higher birth weight, and indirectly with longer gestation for pregnant women regardless of the level of stress [25]. A sense of personal mastery may represent a protective resource related to better mental and physical health among low-income Hispanic women by: a) earlier entry into prenatal care, b) adherence to prenatal care advice, and c) better neonatal outcomes [26].

Corticotrophin Releasing Hormone (CRH) has been intensely studied in relation to stress, coping and the neuroendocrine system. CRH plays a primary role in initiating and controlling the biological stress response [27]. In a recent review, scientists [28] concluded that CRH dysfunction is related to depression and anxiety disorders, thus playing an important role in behavioral adaptation and maladaptation in response to stress. CRH is produced by the placenta and maternal hypothalamus during pregnancy and is thought to play an important role in maintaining pregnancy and the length of gestation [29, 30]. Maternal plasma levels of CRH have been shown to be higher mid-gestation in women who have PTB as compared to those who deliver at term [31]. Additionally, Sandman and colleagues [32] found that early rises in cortisol at 15 weeks predicted later increases in CRH at 31 weeks gestation, indicating initial exposure to stress signals in the placenta resulted in higher CRH and a greater risk of PTB. Maternal or fetal stress conditions may stimulate CRH resulting in the onset of labor. Physiological stress, such as that caused from infections, may also be part of activation of placental CRH pathways, but the role of CRH in response to infection remains elusive [33].

No studies have assessed the joint impact of depression, coping, and mastery as risk factors for PTB. We predicted that the combination of the three psychological variables would better predict and explain the risk of PTB than if used each alone. Therefore, the aim of this analysis was to identify risk profiles for PTB in Hispanic women based on psychosocial factors (depression, mastery, and coping) and to evaluate the associations of psychosocial factors with maternal clinical and socio-demographic characteristics, a biological measure (CRH), and PTB.

## Methods

Data for this analysis were obtained as part of a larger study of immune, endocrine and psychological factors (based on the framework of psychoneuroimmunology or PNI) thought to predict PTB in Hispanic women. The study used a prospective, observational design with a convenience sample, conducted from 2008 to 2012. A convenience sample may be defined as a sample of the population that is readily available or close at hand. Participants included Hispanic women who were self-pay, or who had Title V insurance (state funding), Medicaid, or private insurance. Inclusion criteria were: ability to read and speak English or Spanish, ages 14–40 years old, carrying one fetus, self-identification as a Mexican or Mexican American Hispanic, and living in the United States more than 10 years. Exclusion criteria included: major medical disorders such as diabetes, chronic hypertension, psychiatric disorders identified during the pregnancy, thyroid disorders, and use of steroids at the time of data collection, fetal anomalies, uterine anomalies, fetal demise, placement of a cerclage, multiple gestations. Participants were not excluded if they had a history of preterm birth. Data were collected at 22 to 24 weeks gestation, shown in previous evidence by these investigators to be a critical window for the development of the neuroendocrine system in the fetus and when maternal stressors may significantly affect placental functioning and fetal development and infant outcomes at birth [34]. Sample size for the original study was calculated based on: a) a combination of PNI theory and preliminary results from the investigators previous work and, b) criteria for number of cases in regression and, c) assurance of needed sample size for structural equation modeling [35]. To achieve a power of .80 a sample of at least 375 was needed [35]; data were collected on 515 women to allow for sufficient power after missing data.

Participants were recruited from six private physician practices and two obstetrical clinics in central Texas and the gulf coast area. Participants were approached by a research associate after the study had been initially introduced by the provider. The study was thoroughly explained by the bilingual research associate and a data collection visit scheduled. The bilingual research nurse obtained consent at the data collection visit after ensuring criteria were met. Consent included review of the prenatal record and medical records at delivery. All participants gave written consent; women under the age of 18 years gave child assent and parental consent was also obtained. The research protocol was approved by the Institutional Review Boards of the University of Texas Medical Branch in Galveston as well as at the University of Texas at Austin.

Data collection occurred between 2 to 4 pm to ensure no confounds of diurnal rhythm of the neuroendocrine system. Length of gestation was confirmed using ultrasound reports obtained at less than 20 weeks gestation, preferably less than 12 weeks gestation, and last menstrual period, to ensure that the proper gestational window was being obtained. CRH specimens were obtained from 5 cc’s venous blood drawn into silicone covered EDTA treated vacutainers for all participants at 22–24 weeks gestation. Demographic data was obtained including age, marital status, and highest year of education, annual income and insurance. The research nurse reviewed the prenatal chart for prenatal complications and infections focusing on the type of infections that the participants had prior or during the time of data collection. Standardized questionnaires were administered; if participants had questions the research nurse asked participants to use their best judgment in answering the questionnaires. The participants provided a urine sample to test for cotinine as a marker of smoking. The research nurse accessed hospital medical records for delivery data for both mother and infant, particularly gestational age.

Depression was measured using the Beck Depression Inventory (BDI) [36], a 21-item multiple choice, self-inventory depression scale. The BDI measures the typical symptoms of depression including pessimism, suicide, irritability, insomnia, fatigue, and changes in appetite [37]. Each item is rated on a 4 point scale ranging from 0 to 3, with 63 being the highest total score. Cronbach’s α for this sample was .89 in both English and Spanish. Cronbach’s alpha, or the coefficient of internal consistency, is an indicator of the reliability of an instrument in a given sample. The BDI has previously been tested in pregnant populations to screen for depression [38, 39].

The Brief Cope is a 28 item standardized questionnaire designed to measure cognitive and behavioral coping. It has 14 subscales, each with 2 items, with answers ranging from 1 “not doing this at all” to 4 “doing this a lot.” The scale has both Spanish and English versions [40]. Description of a factor analysis for the results from this study has been published [20] indicating two major factors, active coping and disengaged coping with Cronbach’s α of.80 average for English and Spanish. The Brief COPE has been successfully demonstrated for use in pregnancy in prior studies by George [41].

Mastery was measured using the 7-item Pearlin Mastery Scale [23]. Two items comprise the control subscale measuring a woman’s beliefs that she has control over events in her environment, while 5 items comprise the fatalism subscale, defined as the degree to which a woman believes that the environment controls her life. The fatalism items were reverse scored so that higher scale scores indicated greater mastery. The internal consistency of the total mastery scale was α = .77 for both English and Spanish. DeSocio has used the Mastery Scale to demonstrate self-agency in pregnancy [42] and Christiaens to assess personal control in pregnant women [43].

Level of acculturation was assessed using the 22-item Multidimensional Acculturation Scale II (MAS II) [44]. The MAS II assesses Mexican American or Anglo American cultural identity as well as English and Spanish proficiency. Each item is on a 6-point scale ranging from 0, not applicable, to 5, very applicable. Higher scores indicate greater language proficiency and cultural identity. A principal components analysis revealed four factors for this instrument: English proficiency, Spanish proficiency, American cultural identity, and Mexican cultural identity [44]. In this sample the Cronbach’s α was .84 for both languages. Two other acculturation measures were used: residence index and generational status. To measure residence index participants responded to the question: “How long have you lived in the United States?” The residence index was determined by subtracting the participant’s age from the question response; smaller differences indicated greater exposure to US culture. Generational status was determined by asking about mothers’ and grandmothers’ birthplace: first generation were participants born in Mexico; second generation were born in the US to mothers born in Mexico; and third generation were born in the US to mothers born in the US but whose grandmothers were born in Mexico.

Other multifactorial etiologies were considered as important related to risk of PTB. We collected data on major sociodemographic variables (i.e. age, marital status, education, income, insurance). We obtained clinical data from prenatal and delivery records on history of PTB, gravidity and parity, and preeclampsia. Data collected about infections were primarily derived from cultures or antibody testing: urinary tract infection, group B strep, multiple sexually transmitted infections (i.e. Chlamydia, gonorrhea, herpes simplex virus, human immunodeficiency virus (HIV), hepatitis A, B or C, human papilloma virus (HPV), syphilis, bacterial vaginosis, and candidiasis). Data about infant outcomes were abstracted from the delivery record: gestational age at birth and infant birth weight in grams. PTB was defined as gestation less than or equal to 37 weeks gestation and LBW was defined as weight less than 2500 grams.

The Sure Step™ One-Step Rapid Nicotine Test from Craig Medical Distribution Incorporation (Cat # NIC-X5C) was used as a one-step immunoassay for the detection of cotinine in urine at a cut-off sensitivity level of 200 ng/ml. CRH was detected by the presence of aprotinin 500 IU/ml by radioimmunoassay (RIA) from Phoenix Pharmaceutical Incorporated (Belmont, CA). The protocol has been previously described [45].

A latent profile analysis (LPA) using MPlus 7.11 [46] was conducted to determine the categorical latent variables representing classes of participants with similar psychosocial factors. We constructed LPA using the BDI, Mastery, and the Brief Cope to create latent classes; a total of 18 variables (4 BDI, 2 mastery, and 12 brief coping sub-scales) were used. A series of latent profile models were developed and compared using the Vuong Lo-Mendell-Rubin Adjusted Likelihood Ratio Test (VLMRT), Akaike information criteria (AIC), and the Bayesian information criteria (BIC). The VLMRT provides p-values by comparing the fit of a target model to a model based on one less class. The non-significant result (*p* > 0.05) of VLMRT indicates one less class model fits better. Smaller values of AIC and BIC indicate an improved model fit.

Socio-demographic characteristics, clinical characteristics, biological measures, and infant outcomes were compared across profile groups. Normal continuous variables were compared using a one way analysis of variance while non-normal continuous variables were compared using the Kruskal Wallis test followed by Bonferroni corrected post hoc comparisons. Categorical variables were compared using Fisher’s exact test. Continuous data were described using mean and standard deviation (SD) while categorical data were described using frequency and proportion; the level of significance was set at p < .05. All the statistical analyses other than LPA were conducted using SAS 9.3.

## Results

Mean age of the subjects was 24.6 (SD = 5.8) years. Average years of education of the subjects was 11.8 (SD = 2.4), while median annual income was $23,500. Mean number of years in USA was 20.4 (SD = 6.8) while mean residence index was 0.85 (SD = 0.25). Mean English proficiency was 25.4 (SD = 6.7) and mean Spanish proficiency was 17.9 (SD = 7.2). Mean American cultural identity was 19.2 (SD = 4.4) while mean Mexican cultural identity was 24.5 (SD = 4.8).

All 515 subjects were included in the LPA. Latent profile models specifying 1, 2, 3, and 4 classes were developed separately and compared. The VLMRT showed that the latent profile model containing 3 profile classes was superior to the 2- profile class model (*p* = 0.01). Although the AIC and BIC for the 4-profile classes model were slightly smaller as compared with the 3-profile class model, the VLMRT indicated no improved fit with the 4-class model as compared with the 3-class model (*p* = 0.15). Thus, the latent profile model with 3 profile classes was considered the best model.

**Fig. 1:**
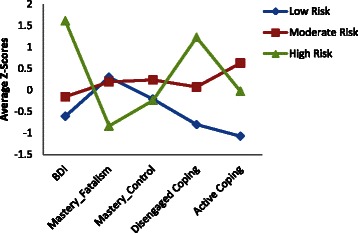
Average Z-scores of psychological variables included in latent profile analysis

Table 1 shows the mean and standard deviation (SD) of each instrument used in the LPA. Figure 1 illustrates the average Z-score of BDI, mastery fatalism, mastery control, behavioral disengagement, and active coping for the three profiles. Profile 1, labeled the “low psychological risk” profile (LPR), represented 30.4 % of the sample: individuals with low levels of depression, relatively high levels of fatalism, and low levels of disengaged coping. Profile 2, labeled the “moderate psychological risk” profile (MPR), represented 52.8 % of the sample: higher depression levels but still within the normal range, high levels on both mastery subscales, moderate levels of disengaged coping and the highest levels of active coping. Profile 3, referred to as the “high psychological risk” profile (HPR), represented 16.7 % of the sample: high levels of depression, relatively low levels of mastery fatalism and mastery positive control, and high levels of disengaged coping and lower active coping scores.

**Table 1:** Distribution of input variables in LPA according to psychological risk groups

Variable	Low risk (*N* = 157)	Moderate risk (*N* = 272)	High risk (*N* = 86)
	Mean (SD)	Mean (SD)	Mean (SD)
Depression BDI-total score	6.12 (4.24)	9.43 (4.48)	22.28 (6.55)
Negative self-attitude BDI subscale	0.93 (1.47)	1.82 (1.90)	8.21 (3.97)
Somatic symptoms BDI subscale	1.94 (1.31)	2.59 (1.40)	4.12 (1.42)
Physical worry BDI subscale	1.33 (0.97)	1.71 (0.98)	2.57 (1.19)
Performance difficulty BDI subscale	1.94 (1.90)	3.31 (2.10)	7.38 (2.51)
Mastery total score	23.4 (3.41)	23.64 (3.15)	19.96 (3.28)
Fatalism subscale of mastery	16.99 (2.80)	16.67 (2.64)	13.59 (2.63)
Control subscale of mastery	6.41 (1.40)	6.98 (1.15)	6.37 (1.17)
The Brief COPE			
Disengaged coping subscales			
Denial	2.54 (0.84)	3.14 (1.36)	4.52 (1.80)
Behavioral disengagement	2.41 (0.72)	2.81 (1.11)	4.43 (1.42)
Substance use	2.02 (0.18)	2.24 (0.86)	3.38 (2.12)
Venting	3.01 (1.02)	4.40 (1.46)	5.62 (1.50)
Self-distraction	3.78 (1.29)	5.43 (1.66)	5.66 (1.58)
Active coping subscales			
Active coping	4.01 (1.49)	6.63 (1.24)	5.34 (1.58)
Use of emotional support	3.73 (1.58)	6.06 (1.58)	5.36 (1.90)
Positive reframing	3.87 (1.26)	6.32 (1.27)	5.14 (1.72)
Planning	3.92 (1.27)	6.49 (1.26)	5.43 (1.68)
Humor	2.90 (1.35)	4.21 (1.77)	4.06 (1.98)
Acceptance	4.12 (1.64)	6.33 (1.43)	5.73 (1.58)
Religion	3.85 (1.56)	5.97 (1.90)	4.97 (1.88)

Table 2 shows the comparison of socio-demographic characteristics and acculturation among the profile groups; age and marital status were associated. Post hoc analysis revealed that the average age was higher in the LPR group compared to the MPR and HPR groups. The LPR group had a larger proportion of married women (56 %) as compared with the MPR (48 %) and HPR groups (27 %). The proportion of married women was also significantly lower in the HPR group compared to the MPR group. All characteristics of acculturation were significantly associated with the risk profile classes except Mexican cultural identity. Post hoc analysis revealed that all characteristics of acculturation in the LPR group were significantly different from the MPR and HPR groups except American cultural identity. No significant differences in acculturation were found between the MPR and the HPR groups. Women in the LPR group had a higher Spanish proficiency than those in the MPR and HPR groups. Other supporting evidence showed that the number of years in the US, residence index, and English proficiency were lower in the LPR group.

**Table 2:** Comparison of socio-demographic characteristics and acculturation according to latent risk profile groups

Variable	Low risk (*N* = 157)	Moderate risk (*N* = 272)	High risk (*N* = 86)	*P*- value
	Mean (SD)	Mean (SD)	Mean (SD)	
Age	25.82 (6.46)	24.32 (5.47)	23.29 (5.25)	0.002
Married - Yes (N, %)	83 (55.70)	124 (48.25)	21 (26.92)	0.0001
Education (years)	11.45 (2.72)	11.99 (2.27)	11.95 (2.27)	0.07
Income (Median, IQR)	21600 (14400, 35000)	25000 (15000, 36400)	20000 (11000, 30000)	0.07^a^
Insurance				0.88
Medicaid (N, %)	79 (50.64)	148 (54.41)	48 (55.81)	
Peri-Chips (N, %)	44 (28.21)	69 (25.37)	24 (27.91)	
Private (N, %)	22 (14.10)	41 (15.07)	11 (12.79)	
Self-pay, or other (N, %)	11 (7.05)	14 (5.15)	3 (3.49)	
Level of acculturation				
Years in the U.S.	19.14 (7.63)	20.82 (6.74)	21.42 (5.26)	0.02
Generation	1.90 (1.05)	2.43 (1.21)	2.44 (1.10)	<.0001
Residence index	0.77 (0.29)	0.87 (0.23)	0.93 (0.17)	<.0001
English proficiency	22.53 (8.50)	26.53 (5.44)	27.01 (4.70)	<.0001
Spanish proficiency	19.42 (6.65)	17.40 (7.30)	16.81 (7.25)	0.005
American cultural identity	18.32 (4.90)	19.86 (4.10)	18.97 (4.18)	0.002
Mexican cultural identity	24.37 (4.55)	24.83 (4.72)	23.69 (5.61)	0.15

^a^Kruskal-Wallis P-Value

There were 57 preterm births: 4 were between 23 and 30 weeks gestation, 7 were between 30 and 34 weeks, 7 were at 34 weeks, 6 were at 35 weeks, and 22 were at 36 weeks to 37 weeks. Only three participants were on antidepressant medications during pregnancy; 2 delivered term and 1 delivered preterm at 27 weeks gestation. Of the 57 preterm births, 36 were spontaneous. There were 8 preterm infants delivered due to the medical complication of preeclampsia or hypertension that did not differ statistically by risk profile. There were 8 preterm births due to preterm premature rupture of membranes, which did not differ statistically by risk profile. There were 2 preterm births from mothers who had gestational diabetes. Finally, there were three preterm infants delivered due to poor biophysical profiles. The maternal and infant delivery outcomes, maternal clinical characteristics, and biological measures were compared across three profile classes as shown in Table 3. The proportion of PTB was 2-fold higher in the HPR group compared to the LPR group. Similarly, the proportion of infants with LBW was more than 2-fold higher in the HPR group compared to the LPR group.

**Table 3:** Comparison of maternal prenatal clinical characteristics, biological measures and infant outcomes according to latent risk profile groups

Variable	Low risk (*N* = 157)	Moderate risk (*N* = 272)	High risk (*N* = 86)	*P*- value
	Mean (SD)	Mean (SD)	Mean (SD)	
Maternal and infant birth outcomes				
PTB- Yes (N, %)	12 (7.74 %)	32 (12.03 %)	13 (15.85 %)	0.14
Type of PTB (*n* = 57 PTBs)				
Spontaneous	11	17	8	0.07
LBW - Yes (N, %)	7 (4.49)	17 (6.42)	9 (10.98)	0.16
Birth Weight (kg) total sample	3296.9 (484.9)	3261.0 (565.0)	3167.6 (559.1)	0.21
Prenatal maternal characteristics total sample				
Start prenatal care (weeks gestation, SD)	9.67 (4.39)	10.15 (4.59)	9.72 (4.43)	0.50
Gravidity	2.94 (1.86)	2.53 (1.51)	2.65 (1.58)	0.04
History of PTB-Yes (N, %)	23 (14.65)	31 (11.4)	14 (16.28)	0.38
Preeclampsia at delivery - Yes (N, %)	2 (1.3)	11 (4.18)	1 (1.23)	0.18
Gestational diabetes - Yes (N, %)	12 (7.79)	18 (6.84)	9 (11.11)	0.42
Infections present (N, %)	38 (26.03)	96 (36.64)	40 (49.38)	0.002
Biological measures at 22–24 weeks gestation				
Cotinine positive -Yes (N, %)	3 (1.92)	9 (3.36)	4 (4.65)	0.45
Corticotrophin releasing hormone: pg/ml (Median, IQR)	19.8 (12.9, 38.1)	27.4 (14.9, 50.7)	23.7 (12.0, 41.7)	0.02^a^

^a^Kruskal-Wallis P-Value

The difference in proportions of infections was statistically significant between the LPR and HPR groups. Almost 50 % of the women in the HPR group had prenatal infections versus 26 % of the women in the LPR group (See Fig. 1). A group B strep positive culture was present in 19 % of the sample. The most prevalent types of infection were sexually transmitted infections (STIs) (6 %). Of the STI’s, chlamydia was most prevalent (3.9 %) and then HPV (1.9 %); only 6 women (1.2 %) had herpes infections and only 1 had gonorrhea (.2 %). Urinary tract infections were documented in 21 cases (4.1 %). There were 9 cases of bacterial vaginosis (2.2 %) and no cases of HIV or hepatitis.

**Fig. 2:**
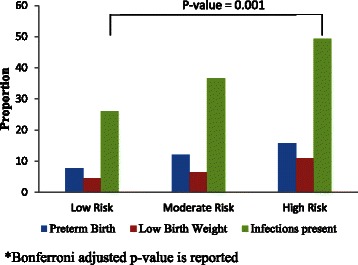
Comparison of preterm birth, low birth weight and infections according to latent profile groups

Average gravidity was significantly higher in the LPR group compared to the MPR group. The mean CRH was higher in the MPR group compared to the LPR group. No significant difference in mean CRH was observed between the MPR and HPR groups. Figure 2, which depicts the distribution of outcomes according to the profile classes, shows the trend in increasingly poor infant outcomes from the LPR to MPR groups and from MPR to HPR groups.

## Discussion

In the current analysis, we found that a combination of psychological factors (depression, coping and mastery) determined by LPA, predicted different profiles of risk for PTB in Hispanic women of Mexican origin. The rate of PTB in the LPR group was 7.74 % (*n* = 157) versus 15.8 % (*n* = 86) in the HPR, double the percentage. The MPR group had a 12 % (*n* = 272) PTB rate, which is similar to the cited 11.6 % PTB for Hispanics in the United States [47]. The HPR group’s 15.8 % approaches the 17.1 % rate of PTB in African Americans [48]. The LPR group’s 7.74 % is consistent with previous research on the Hispanic paradox [49, 50], whereby more recent Mexican immigrants have more favorable health outcomes. The combined measures with acculturation measure using depression, mastery and coping give more data on risk than just using one measure such as depression. The combined measures also provide targets for interventions (mastery and coping) that may affect depression.

The sociodemographic characteristics of the HPR included a mean income just above the 2012 federally defined poverty level of $23,050 [51]. In addition, the HPR had the highest rate of single mothers and the youngest women. These sociodemographic characteristics are consistent with Reedy’s premise that it is a combination of factors that puts women at risk for PTB [52].

Conversely, when considering the demographic and self-report measures of acculturation, the LPR group with the lowest risk profile for PTB, had the most women who were in the United States the shortest time, were more proficient in Spanish and identified less with American cultural ways. This finding is consistent with recent studies supporting the hypothesis that Mexican born women and women with stronger Mexican affiliation have a perinatal advantage over women of Mexican origin with higher levels of acculturation [53, 54].

The disadvantage of greater acculturation is seen also in the HPR group with double the percentage of total infections noted at the time of data collection, a finding consistent with a large body of evidence supporting the role of infections and PTB [55]. The high rate of infections may also be related to life style factors associated with being single and/or having lower socioeconomic status, characteristics also found in the HPR group. The LPR group with the lowest risk of PTB and the least acculturated women, also tended to be older and married, and had the lowest percentage of total infections.

As hypothesized, CRH was highest in the two highest risk profiles. This finding is consistent with the finding of greater levels of acculturation in these profile groups and may be a reflection of behavioral maladaptation in relationship to stress. The MPR group had the highest levels of CRH and the highest rate of preeclampsia. CRH has been shown to be greatly elevated with preeclampsia [33], thus a possible explanation for this finding. Our findings related to CRH at 22–24 weeks predicting PTB differ from those results of Sandman that indicated that the effect of CRH is restricted to weeks 26–31 [32]. However, our findings corroborate Sandman’s findings that CRH levels are primed in relationship to stress and are thus higher and go up faster in women who will deliver preterm.

The HPR group had the highest depression scores and a high mean CRH. This finding is consistent with previous research indicating CRH dysfunction and depression are linked [28, 29]. This finding also confirms finding that have been suggestive of the potentially important role CRH may play in the biological cascade leading to PTB [30]. The mean depression score in the HPR group indicates moderate depression [56]. This finding corroborates findings showing higher levels of maternal depression escalate the risk of PTB [11, 16]. This result is also important in light of the effects of maternal depression on fetal development and early child development [57] and the increased risk for postpartum depression if prenatal depression is present [16]. The results regarding coping are consistent with the literature that first generation Hispanics, notably seen in the LPR, used less active coping and had the lowest overall scores for coping [58]. The MPR group, with the highest coping scores in the active subscales of all three profiles, may be best protected from PTB with the pattern of depression scores seen in this group reflective of normal ups and downs of moods [37]. The HPR group had the highest scores for disengaged coping, a finding associated with poor psychological wellbeing [21, 22] and consistent with prior research [17]. Additionally, the avoidance coping scores for this group are also consistent with being single [18]. The HPR group also had the lowest mastery scores. Low mastery has been associated with depressive symptoms and an increased risk of PTB [10].

### Strengths and limitations

This study had several notable strengths including the large and homogeneous Mexican American sample from four generations. Reliable measures were used in both Spanish and English. CRH was run by RIA, with greater sensitivity as an assay. Multiple confounding measures were controlled for.

The study also had limitations. The sample was one of convenience. Second, a cross sectional one-time measurement was done at 22–24 weeks, so causality cannot be determined, nor can changes or longitudinal trends in CRH be determined. Finally, the sample focused on Hispanics of Mexican origin, thus it is not known if these risk profiles are appropriate for Hispanics from other countries, such as Puerto Rico or the Dominican Republic.

## Conclusions

The results from this study suggest both a need and a possible basis for risk screening early in pregnancy in Hispanic women of Mexican origin. When coupled with CRH as a biomarker, risk screening might allow focused attention for women most at risk for PTB. Further refinement of the risk profiles for predicting PTB is needed by using a stronger design, such as a prospective longitudinal cohort. Determination of the most predictive questions to use for screening is also needed. Research is also needed to determine a threshold of CRH for PTB risk in Mexican American Hispanics. Over time, research should focus on the development of non-pharmacological interventions that target amenable factors in the most at-risk profiles. In particular, interventions to reduce depression, increase mastery and improve active coping are all indicated. Future research should include use of risk profiles with targeted interventions tailored to the Hispanic culture.
